# On Having No Head: Cognition throughout Biological Systems

**DOI:** 10.3389/fpsyg.2016.00902

**Published:** 2016-06-21

**Authors:** František Baluška, Michael Levin

**Affiliations:** ^1^Department of Plant Cell Biology, IZMB, University of BonnBonn, Germany; ^2^Biology Department, Tufts Center for Regenerative and Developmental Biology, Tufts UniversityMedford, MA, USA

**Keywords:** aneural, cognition, plants, bioelectric signaling, computation, memory, information, learning

## Abstract

The central nervous system (CNS) underlies memory, perception, decision-making, and behavior in numerous organisms. However, neural networks have no monopoly on the signaling functions that implement these remarkable algorithms. It is often forgotten that neurons optimized cellular signaling modes that existed long before the CNS appeared during evolution, and were used by somatic cellular networks to orchestrate physiology, embryonic development, and behavior. Many of the key dynamics that enable information processing can, in fact, be implemented by different biological hardware. This is widely exploited by organisms throughout the tree of life. Here, we review data on memory, learning, and other aspects of cognition in a range of models, including single celled organisms, plants, and tissues in animal bodies. We discuss current knowledge of the molecular mechanisms at work in these systems, and suggest several hypotheses for future investigation. The study of cognitive processes implemented in aneural contexts is a fascinating, highly interdisciplinary topic that has many implications for evolution, cell biology, regenerative medicine, computer science, and synthetic bioengineering.

## Introduction

Survival in a complex, dynamic, and highly competitive environment requires biological systems to make numerous decisions with respect to possible activities (Conrad, 1996; Holcombe and Paton, 1998). Evolutionary pressure to optimize decision-making has led to the inevitable exploitation of past history (memory) and information processing (computation). Importantly however, decisions are made at every level of biological organization. For example, multicellular organisms, such as animals and higher plants, exhibit multilayer complex goal-directed behaviors also at their cellular and subcellular levels. Underlying physiological systems must maintain homeostasis and predict future conditions (Freddolino and Tavazoie, 2012) in the face of unpredictable changes in environmental conditions, while cells must coordinate their activity in an exquisite 3-dimensional ballet of embryogenesis and complex organ regeneration. At the extremes of the scale of organization, dynamic self-organizing subcellular components like cytoskeleton and molecular networks (Albrecht-Buehler, 1985; Craddock et al., 2012; for plant cells see Volkmann and Baluška, 1999; Barlow and Baluška, 2000) and colonies of organisms (Shapiro, 1998; Couzin, 2009) perform similar functions in their own contexts. Here, “cognition” refers to the total set of mechanisms and processes that underlie information acquisition, storage, processing, and use, at any level of organization (Lyon, 2015).

Memory is an essential component of these processes, at all levels. For our purposes, memory can be defined as experience-dependent modification of internal structure, in a stimulus-specific manner that alters the way the system will respond to stimuli in the future as a function of its past. This requires a labile yet stable medium, to provide the necessary latency. The process may or may not involve a degree of intelligence, in the sense of the ability to compress prior stimuli into informationally-compact representations (inference). In essence, sensory memory is a message to one’s future self – a view reminds us that memory is thus another instance of biological communication (which, as exchange of signals, is ubiquitous among all levels of biological organization). Put this way, we can see that in principle many biological mechanisms can be exploited for this purpose. The updates in the configuration (or state) of a system, as occurs also during sensory memory formation in all organisms, is formally known as “computation” in computer science.

These concepts are quite general. However, outside of the unconventional cognition community (Calvo and Baluška, 2015) or biological computation community (Adamatzky et al., 2008), it is widely assumed that memory is the exclusive province of brains, or even complex animals. Older work exploring these issues in plants (reviewed by Gremiaux et al., 2014), non-neural somatic tissues (Mackie, 1970), and even inorganic media (Bose, 1926), have been largely forgotten in favor of the remarkable advances in recent cognitive neurosciences with their focus on the brain. Nevertheless, plant cells are known to be able to use action potentials to control their movements and behavior since times when Charles Darwin and Jagadis Chandra Bose turned their interest toward plants (Darwin, 1880; Shepherd, 2005; Baluška et al., 2009a). Currently, surprisingly, higher plants are emerging as behaviorally active organisms, enjoying bio-communication and showing plant-specific cognition and intelligence (Trewavas, 2005, 2014; Karban et al., 2014a,b; Calvo and Baluška, 2015; Calvo, 2016).

**FIGURE 1:**
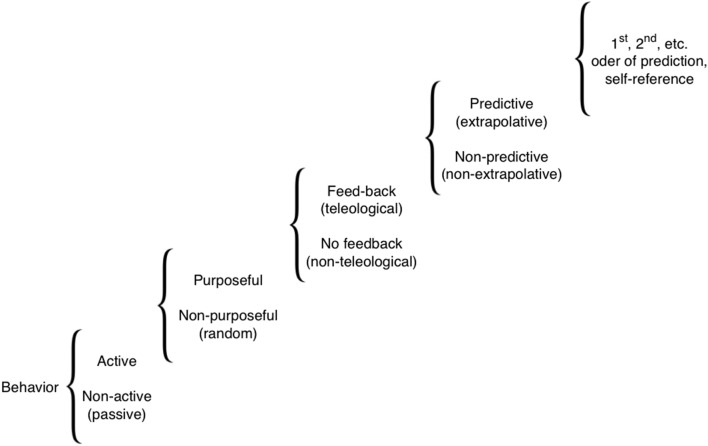
**A scale of cognitive levels**. There are many types of cognition, from simple reflexive behaviors all the way to systems that can internally model themselves and their environment to compute counterfactuals and make complex choices. Various biological systems can be considered cognitive to the extent that modeling them at one of these levels provides improved (more accurate or efficient) predictive and control capabilities. Reproduced from Rosenblueth et al. (1943).

Here, we survey a wide-ranging literature on memory and sensory systems-based cognition in organisms (biological systems) lacking animal/human-type brains. Our goal is to acquaint readers interested in cognition with numerous aneural model systems in which this subject can be pursued, and to draw the attention of bench biologists working on those systems to cognitive, information-focused perspectives on the mechanisms they are studying. Importantly, in discussing cognitive performance in the various systems, we do not mean the full-blown human-like cognitive performance, or human-type of self-awareness and consciousness. We are avoiding issues of the ‘*Hard*’ problem of cognitive science, and do not claim anything like higher-order symbolic representations. Our definition is purely functional and minimalist (Calvo and Baluška, 2015), drawing attention to the similarities in computational tasks performed by diverse biological systems, at all levels of complexity, other than animal and human brains. **Figure 1** illustrates the full spectrum of cognitive levels and capabilities upon which the various systems we discuss can be placed (Rosenblueth et al., 1943). Our review begins with a consideration of the familiar substrate of cognition: neural dynamics, and of mechanisms that blur the boundaries between neural and non-neural cell functions. We then proceed through progressively more divergent cognitive systems, considering molecular networks, single cell behaviors, networks of cells in various tissues, and organism-wide information processing during regenerative repair. We conclude with some common threads of cognition across levels of organization, which suggest a unified perspective on these highly diverse systems.

## Neurons: Their Powers, Evolutionary History, and Beyond

Recent work has begun to encompass cognition in *ex vivo* systems, with studies that have shown training and learning in cultured minimal neural networks (DeMarse and Dockendorf, 2005; Dranias et al., 2013; Pimashkin et al., 2013). Even *in vivo*, it is increasingly recognized how much processing happens before signals get to the brain of the central nervous system (CNS); a recent example is the discovery that neurons in the skin perform edge detection (Pruszynski and Johansson, 2014).

Importantly, CNS neurons do not embody cognition due to any magical, unique property. Their computational powers derive from the dynamics of networks of linked elements that propagate and integrate signals, and the ability to alter connectivity among those elements (network topology) based on prior activity. In fact, these basic properties are present in biological systems at many complexity scales (from subcellular protein networks to coupled tissues). Might they too underlie some aspects of cognitive-like information processing? Indeed, neurons did not invent their special tricks – they merely optimized them for speed to drive adaptive behavior. These functions, and the molecular mechanisms that implement them – ion channels, electrical synapses (gap junctions), and neurotransmitter molecules are all ancient (Goldsworthy, 1983; Baluška, 2010; Brunet and Arendt, 2016; Moroz and Kohn, 2016). Neural networks evolved from far older signaling pathways that orchestrated development, physiology, and other cellular functions long before the CNS arrived on the evolutionary scene (Buznikov et al., 1996; Levin et al., 2006; Keijzer et al., 2013). Already simple cells of bacteria enjoy sensory systems feeding into cognitive-behavioral circuits and showing many other neural features (Miller and Koshland, 1977; Koshland, 1980; Lyon, 2015). Electrical long-distance signaling and information exchange via spatially propagating waves of potassium is synchronizing bacterial biofilms (Beagle and Lockless, 2015; Nunes-Alves, 2015; Prindle et al., 2015). Integrated bacteria within the biofilm community appear to act as some kind of ‘microbial brain’. Obviously, the neuronal communication has bacterial origins (Baluška and Mancuso, 2009).

The main principles by which neural networks store and process information – plasticity, excitability, and experience-dependent change (Daoudal and Debanne, 2003) are readily applicable to numerous cell types. Indeed, the computational powers of glia and other non-spiking cells in the brain are increasingly appreciated in their contributions to mammalian cognition and intelligence (Oberheim et al., 2009; Goldman et al., 2015). Astrocyte networks perform computations (Schummers et al., 2008), and models of memory have long been proposed that rely on non-spiking neurons (Aur, 2012), revealing that neural-specific, discrete action potentials are not a pre-requisite for memory dynamics.

At the same time, tissues other than neurons are able to conduct the kind of signaling impulses that are considered the *sine qua non* of cognition. For example, excitation and impulse propagation have been shown in skin (Roberts and Stirling, 1971; James and Soffe, 2011). The evolution of neurons from excitable precursors has been reviewed elsewhere (Mackie, 1970; Baluška and Mancuso, 2009; Baluška, 2010; Moroz and Kohn, 2016), as have the many similarities between neurons and other cell types (Bharti and Arnheiter, 2005; Yaar and Park, 2012). In this overview, we cast our net even broader, examining examples of cognition outside of the CNS domain of life (Calvo and Baluška, 2015; Lyon, 2015; Calvo, 2016), with or without spiking, in cellular networks of complex metazoans, or within single-cells. We also review some of the mechanisms that underlie this cognition which is inherent to cellular life at all levels of biological complexity, and suggest a few novel experimental directions that may exploit the deep lessons suggested by the ubiquitous nature of aneural cognition.

## Crossover Between Non-Neural and Neural Mechanisms

The interplay of neural and non-neural signaling has been shown in several regenerative systems. Neural inputs are required for amphibian limb regeneration (Singer, 1952; Kumar and Brockes, 2012), although curiously, this is not a hardwired requirement but must be learned: limbs that grew without the presence of a nerve later do not require nerve to regenerate, unlike normal limbs. This phenomenon has been termed “nerve addiction” (Yntema, 1959a,b; Filoni et al., 1995), extending the principle of experience-dependent long term change to limb regeneration. Neural inputs are also needed for maintenance of tissue structure in the rodent tongue (Takeda et al., 1996; Sollars et al., 2002), suppression of tumors in rabbits (Pawlowski and Weddell, 1967; Pawlowski, 1970), and regulation of specific pattern in distal tail regeneration in amphibia (Mondia et al., 2011).

Neural and non-neural information systems must cooperate especially when pattern formation and memory intersect. A unique model system for these studies is planaria, the free-living flatworm (Gentile et al., 2011); this is a unique model system that regenerates every part of its body (Reddien and Sánchez Alvarado, 2004) and also possesses a true centralized brain (Sarnat and Netsky, 1985; Pagán, 2014) and learning capabilities (Wells, 1967; Sheiman and Tiras, 1996; Nicolas et al., 2008). In this model species, the dynamics of behavioral memory can be studied during complete brain regeneration [in the axolotl, this can also be done, albeit with only partial brain regeneration (Pietsch and Schneider, 1969)]. Classical studies (McConnell et al., 1959; Corning, 1966), as well as more recent work performed using automated analysis methods (Shomrat and Levin, 2013), showed that memories in planaria survive decapitation – tail fragments trained on a task regenerate brains and then show evidence of recall of the original information. This requires the body to store learned information and imprint it on the nascent brain after it is rebuilt. The mechanisms of this interaction are completely unknown, but offer an unprecedented opportunity to study transfer between neural and somatic memory systems.

## Molecular Mechanisms of Non-Neural Cognition

Memory, and often the intermediate processes of computation, requires that “stimuli produce a permanent record written on the irritable substance” (Semon and Simon, 1921). What underlying mechanisms have been implicated in non-neural memory and related processes?

One of the best candidates for mechanisms underlying information processing at the single cell level is the cytoskeleton (Albrecht-Buehler, 1985; Craddock et al., 2010; Sahu et al., 2013), which has all of the necessary properties: it is a large, complex structure that is readily modified by a variety of molecular pathways (writing data), is interpreted by numerous motor proteins and other machinery (reading data), and implements a rich set of discrete transition states that could implement computational operations (Hameroff and Watt, 1982; Lahoz-Beltra et al., 1993; Volkmann and Baluška, 1999; Craddock et al., 2012). The cytoskeleton has long been a favorite locus of information storage and control in single-celled organisms, where it regulates behavior (Eisenstein, 1967; Hamilton, 1975) and serves as a non-genomic repository for permanent somatic changes such as cell surface chirality changes (Nelsen et al., 1989). The dynamic actin cytoskeleton behaves as excitable medium (Khan et al., 2012).

Another medium for information processing is within chemical networks, such as reaction-diffusion (RD) dynamics that underlie pattern formation in embryogenesis (Kondo, 2002; Kondo and Miura, 2010; Raspopovic et al., 2014). Recent work has revealed that RD systems and similar excitable chemical media can be designed so as to execute specific computations, and are being used for the design of minimal cognition controllers (Dale and Husbands, 2010) and other kinds of computation including planning (Adamatzky et al., 2003; Adamatzky et al., 2008; Costello et al., 2009). Remarkably, it was shown long ago (Rosen, 1968) that Rashevsky’s 2-factor systems (a model for neuronal excitation) is formally equivalent to Turing’s RD scheme for self-organizing morphogenesis (Turing, 1952). Grossberg then described extensive parallelism between signal processing in chemical gradients during development and neural memory and visual processing (Grossberg, 1978). RD systems are Turing-complete (Scarle, 2009) and support semantical interpretations (Schumann and Adamatzky, 2009), making them an excellent candidate for complex computations. Recent work used *in silico* evolution of chemical networks to show that simple, plausible reactions can be found which perform associative learning and Bayesian behavior which includes memory traces (McGregor et al., 2012). These data are especially exciting in that they imply that associative learning can readily evolve in metabolic, gene regulatory, or intracellular signaling networks.

The transcriptional control machinery that guides embryogenesis has also been modeled as cognitive processes. Gene regulatory networks can be modeled as neural networks (Watson et al., 2010), with genes representing nodes and functional links representing inductive or repressive relationships among those genes. That landmark study showed that changes to the connections in the regulatory net represent a kind of Hebbian plasticity (as genes whose expression is up-regulated in specific environments tend to become co-regulated and thus expressed together). In part due to this fire-together-wire-together process, a GRN will develop an associative memory of phenotypes selected in the past. This view sheds important light on the relationship between homeostasis and evolvability and shows that a transcriptional network can develop memory and recall capabilities often thought to be reserved for classical cognitive systems. As a consequence of memory, genetic networks can exhibit predictive ability, enabling anticipatory behavior with respect to physiological stimuli (Tagkopoulos et al., 2008). A similar result was obtained for protein networks, showing that signaling via the tumor suppressor P53 could be modeled as a neural net (Ling et al., 2013), while MAP kinase pathways implement specific decision-making processes (McClean et al., 2007). Embryos make use of genetically encoded cellular memory, for example in the case of HOX gene expression patterns, which constitute a form of positional memory – “an internal representation by a cell of where it is located within a multicellular organism” (Chang et al., 2002; Rinn et al., 2006; Wang et al., 2009), and hysteresis in Hedgehog protein signaling (Balaskas et al., 2012), all of which are used to guide the subsequent activity of cells as a function of prior “experience”.

Additional memory media include the extracellular matrix (Becchetti et al., 2010; for plant cell walls see Humphrey et al., 2007; Seifert and Blaukopf, 2010; Hamann, 2015) and chromatin complex markings (Francis and Kingston, 2001; Maurange and Paro, 2002; Ringrose and Paro, 2004), both of which are ideal media for recording traces representing specific environmental and/or physiological events. These are examples of internal stigmergy – activity that leaves traces in a labile intracellular or extracellular medium which can be read as memories in the future by cells making decisions for migration, differentiation, apoptosis, or signaling (Theraulaz and Bonabeau, 1999; Ricci et al., 2007).

Importantly, many cell types communicate electrically, not just excitable nerve and muscle (McCaig et al., 2005; Levin, 2007a,b, 2012a; Bates, 2015). Recent molecular data show that developmental bioelectricity is an important modality by which cell networks process information that instructs patterning during regeneration, development, and cancer suppression (Levin, 2014a,b,c). Thus, one obvious candidate for cognition outside the brain is via the same mechanism used in the brain – bioelectrical networks (Levin and Stevenson, 2012; Mustard and Levin, 2014). Indeed it is likely that the processing in the brain is a direct extension (and speed optimization) of far older mechanisms used originally for morphogenesis (Buznikov and Shmukler, 1981; Levin et al., 2006). Developmental bioelectricity in animal systems features slowly-changing, continuous voltage changes as opposed to millisecond discrete (binary) spiking usually studied in the brain. However, the brain also includes non-spiking neurons (Victor, 1999) that have computational compartments similar to the membrane voltage domains observed in embryonic and other non-neural cells (Levin, 2007b; Adams and Levin, 2012). It has recently been proposed (Levin, 2012b, 2013; Mustard and Levin, 2014) that non-neural tissues support the same two types of plasticity as seen in the brain: changes of connectivity via electrical synapses (gap junctions) which corresponds to synaptic plasticity, and changes of ion channel function which corresponds to intrinsic plasticity (Marder et al., 1996; Turrigiano et al., 1996; Daoudal and Debanne, 2003). In addition to computation via changes in resting potential, which is a primary regulator of pattern memory in embryogenesis and regeneration (Adams, 2008; Funk, 2013; Levin, 2014b), as well as of processing in the brain (Sachidhanandam et al., 2013; Yamashita et al., 2013), ion pumps such as the ubiquitous sodium-potassium ATPase, have been suggested as computational elements (Forrest, 2014).

Most of these bioelectrically active systems are based on ion dynamics at membranes which modify bioelectric fields via activities of ion channels and transporters (Taylor, 1974; Wayne, 1993, 1994; Hille, 2001). These membrane-associated electric fields feed-back on membranes and associated proteins (Jaffe, 1977; Tsong and Astumian, 1986; Westerhoff et al., 1986; Bezanilla, 2002, 2006, 2008). They also control endocytosis and vesicle trafficking (Antov et al., 2005; Baluška and Wan, 2012). Relevantly, even biochemical reactions are under electric control (Aragoněs et al., 2016; Xiang and Tao, 2016), as is transcription (Pai et al., 2015b) and chromatin modification (Carneiro et al., 2011; Chernet and Levin, 2014).

## Cognitive Capabilities of Single Cells

While the dominant model of neural-based cognition relies on the signaling dynamics among networks of neurons, it’s becoming increasingly appreciated that single neurons can execute subtraction, addition, low- and band-pass filtering, normalization, gain control, saturation, amplification, multiplication, and thresholding with respect to the input-output relations they implement (Koch and Segev, 2000). Memory and computation is thus not exclusively a multi-cellular phenomenon, and is not restricted to somatic neural cells. Recent computational studies have revealed conditions under which cells expressing ion channels can keep a stable memory with respect to resting potential, and these conditions do not specifically require neuronal cell identity – they can be fulfilled by numerous cell types, somatic as well as free-living (Ramanathan and Broach, 2007; Cervera et al., 2014; Law and Levin, 2015).

The amoeba of *Dictyostelium discoideum* migrate by extending pseudopods in an alternating pattern. The specific pattern of the pseudopods’ zig-zag behavior was recently shown to be predictable by viewing the cell surface as an excitable medium. In this model, the appearance of a pseudopod makes the local cortex temporarily more excitable (a kind of potentiation), while globally new pseudopods are inhibited. This model thus includes a memory of previous pseudopod locations, and quantitatively fits data from cell tracking experiments and the known chemotactic sensitivity of these cells (Cooper et al., 2012).

Budding yeast also keep a history which influences their future behavior – a memory of past events. They avoid pheromone-induced cessation of cell cycle after a deceptive mating attempt (failure to reach a putative partner cell within a specific time period). The mechanisms of this are beginning to be unraveled (driven by the dynamics of the maternally segregating G1/S inhibitor Whi3), and the authors term the macromolecular assemblies that mediate this memory “mnemons”, cellular structures that encode previous environmental conditions (Caudron and Barral, 2013). With respect to the search for the molecular substrate of specific memories, this yeast work may be ahead of similar efforts in the brain (Ungar, 1972, 1974a,b).

The flexible and versatile responses of bacteria to their environment has drawn significant attention of synthetic, molecular, and evolutionary biologists, as well as those interested in unconventional computational media (Miller and Koshland, 1977; Koshland, 1980; Ben-Jacob, 2009; Norris et al., 2011). Single bacteria are able to migrate toward beneficial targets, and away from noxious stimuli. The control algorithm for this behavior has long been the subject of investigation, with respect to the short-term memory needed for following gradients (Vladimirov and Sourjik, 2009) as well as “infotaxis” policies that do not require gradient sensing (Vergassola et al., 2007). Especially exciting are the recent findings that bacterial communities (biofilms) process information and make decisions about nutrient distribution and metabolism as an integrated whole, using ion channels (Prindle et al., 2015) and a kind of volume transmission as occurs in the brain (Agnati et al., 2006; Fuxe et al., 2013; Zhang et al., 2013). Ciliates (protozoa) exhibit learning and a form of memory, which even survives loss of nuclei and some cytoplasm (Gelber, 1962; Applewhite et al., 1969; Hamilton, 1975; Clark, 2013). The mechanism is unknown, but may involve electrical signaling (Applewhite, 1972; Kunita et al., 2014).

In addition to cells that make their living independently, single somatic cells from metazoan organisms also exhibit memory and decision-making (Albrecht-Buehler, 1985) during directed steering (Albrecht-Buehler, 1982) – a capability that also extends to cell fragments (Albrecht-Buehler, 1980) and even human sperm that adjust their flagellar beat to reach the egg via calcium-dependent tracking of chemical attractants (Alvarez et al., 2013).

The immune system has long been a paradigm of pattern recognition and classification (Carter, 2000). While the mainstream view of immune function is that of an evolutionary system driven by selection, a cognitive perspective has been proposed (Cohen, 1992a,b) as an alternative theoretical framework for understanding the body’s remarkable ability to distinguish self from non-self and adapt via immunological memory. It is interesting that the converse proposal has been made as well, to understand brain dynamics via a selectionist model (Fernando et al., 2012). Thus, in some sense, evolutionary and cognitive dynamics could be parallel (isomorphic?) ways to explain complex systems. If true in general, it may have significant implications for evolutionary theory.

An interesting kind of cognitive process is revealed by drug addiction. The increased tolerance with exposure is desensitization (one kind of basic memory element). Drug addiction reactions have been shown in somatic mammalian cells in culture (Corssen and Skora, 1964; Manner et al., 1974; Higgins et al., 1978), suggesting that this form of memory is not always a body-level phenomenon that necessarily involves the brain.

## Slime Molds: Between Unicellular Life and Metazoan Bodies

*Physarum polycephalum* is a slime mold that has been extensively used in studies of biological information processing (Nakagaki et al., 2004; Saigusa et al., 2008). By computing optimal paths for nutrients throughout its syncytial body, the organism can implement behavior that solves challenging spatial optimization problems, such as solving mazes and finding efficient highway layouts (Nakagaki et al., 2004; Saigusa et al., 2008; Adamatzky and Alonso-Sanz, 2011). This organism shows how the internal dynamics of morphogenesis, even at this primitive step toward a multicellular bodyplan, can implement decision-making and computation. It is particularly interesting that in this system, a kind of variational (least-action, or minimization) principle is explicitly implemented by a biological medium (Friston, 2010; Friston et al., 2015), providing a much-needed “base case” for starting to understand the common features of goal-directed activity across levels of organization from cells to body structures to organism behavior.

*Physarum* also shows evidence of memory. In their study of the Traveling Salesman Problem (requiring an optimal strategy for connecting regions in space), Zhu et al. (2013) found that when two individuals were created by dividing one individual, they remained correlated in their exploration even though they were spatially separated, suggesting the presence of a long-term memory in the intrinsic dynamics.

## Cognition in Plants

Although plants are still considered generally to be outside of neuronal and cognitive organisms, due to their lacking of animal-type of neurons and brains, plant cells have many features which are considered neuronal, including plasma membrane excitability supporting action potentials, acentriolar microtubules, motile Trans Golgi Networks, and synaptic-like actin-enriched cell-cell adhesion domains (Wayne, 1993, 1994; Barlow and Baluška, 2000; Baluška et al., 2003, 2005, 2008, 2009b; Baluška, 2010). Especially cells in root apices are very active in these neuronal-like activities and act as *brain-like* command centers (Baluška et al., 2004, 2009a,b, 2010; Baluška and Mancuso, 2009, 2013), navigating growing roots in their search for water and mineral nutrients in soil, and active root avoidance or escape from toxic, stressful and dangerous situations (Burbach et al., 2012; Yokawa et al., 2014; Yokawa and Baluška, 2015, 2016).

The classic studies on plants showing animal-like features and activities were accomplished more that 150 years ago by Charles Darwin, assisted with his son Francis Darwin, and Claude Bernard (Darwin, 1880; Bancroft and Richter, 1930; Perouansky, 2012). Later, Jagadis Bose accomplished his sophisticated experiments on plants, confirming and extending the previous results obtained by Charles Darwin and Claude Bernard (Shepherd, 2005). Despite the fact that plant action potentials are known for more than 150 years now, and these are known to control many plant processes (Wayne, 1993, 1994; Masi et al., 2009; Volkov et al., 2010; Sukhov et al., 2011; Böhm et al., 2016; Hedrich et al., 2016), plant action potentials are still ignored by the mainstream. For example, there is no single mention of plant action potentials in the book Plant Physiology by Lincoln Taiz, which represent the most accepted view of plants in biology (Taiz, 2010).

Claude Bernard performed many anesthetic experiments. He expanded experimental materials from animals to plants. He showed that the Mimosa plant (*Mimosa pudica*), closing leaves upon touch, was unresponsive when exposed to a diethyl ether atmosphere which immobilized mice. Claude Bernard arrived at the conclusion that plants and animals share a common biological principle that is disrupted by anesthetics. He hypothesized that similarly as animals, also plants are able to actively sense their environment. He called this ability plant “sensitivity”. In order to test his ideas, he performed anesthesia on plants and the results of these experiments were presented in 1878 in “*Leçons sur les phénomènes de la vie communs aux animaux et aux végétaux*” (Bernard, 1878; Bancroft and Richter, 1930). Later, sensitivity of plants to anesthetics was confirmed not only for Mimosa and Dionea, but also for many other plants (Milne and Beamish, 1999; De Luccia, 2012; Gremiaux et al., 2014).

Similarly as neurons, plant cells are excitable and plant-specific action potentials serve for long-distance communication and integration of plant bodies. Action potential also control rapid plant organ movements such as closing the Dionea traps or touch-induced movements of Mimosa leafs (Volkov et al., 2010; Böhm et al., 2016; Hedrich et al., 2016). Our preliminary data with Dionea traps suggest that anesthetics block action potentials (Yokawa et al., in preparation). Moreover, action potentials control also nutrient transporters in Dionea prey-stimulated traps (Böhm et al., 2016; Hedrich et al., 2016). In the root apex, the transition zone is very active not only in electric activities (Masi et al., 2009), and synaptic-like cell-cell communication (Baluška et al., 2003, 2004, 2005, 2009a,b, 2010; Baluška and Mancuso, 2013), but also in sensory-based control of root growth navigation associated with high electric activity. Root apex navigation is based on complex computations as roots sample continuously huge amounts of abiotic and biotic information from their environment in order to find water and nutrient rich zones in soil; and to avoid dry, toxic and dangerous zones. Our data suggest that root navigation is controlled via computations accomplished at the root apex synapses and associated with electric activities (Masi et al., 2009).

Plants are emerging as excellent biological computational systems. For example, leaves maintain stable temperature near their surfaces despite large fluctuations of temperature in the atmosphere (Helliker and Richter, 2008; Pincebourde and Woods, 2012). They relay in leaf stomata which acts as plant thermostats tissue, with individual stomata acting as autonomous units showing collective behavior (Hetherington and Woodward, 2003; Peak et al., 2004). In the case of plant leaves, stomata are simultaneously the sensors of external information, the processing units that calculate gas exchange rates and sensitively regulate their controls. Plants solved the dilemma of optimal gas exchanges via elegant parsimonious computational techniques in which input, output, and processing are all accomplished by using the same hardware.

Additional nice examples of plant computation include the ability of plants to compute starch synthesis and degradation rates (Scialdone et al., 2013; Webb and Satake, 2015), root apex computation of numerous abiotic and biotic parameters to navigate optimally root growth in complex environment of patchy soil (Baluška et al., 2009a,b, 2010; Masi et al., 2009; Baluška and Mancuso, 2013), as well as computations accomplished via Dionea leaf traps (Volkov et al., 2010; Böhm et al., 2016). Action potentials are relevant for most (perhaps all) of plant-specific computations (Masi et al., 2009; Volkov et al., 2010; Böhm et al., 2016; Hedrich et al., 2016).

In the root apex transition zone, cells and their membranes oscillate in almost all their activities (Baluška and Mancuso, 2013). These root apex transition zones resemble presomitic mesoderm segmentation clocks underlying vertebrate embryo segmentation (Moreno-Risueno et al., 2010; Traas and Vernoux, 2010; Moreno-Risueno and Benfey, 2011).

## Animal Cell Physiology as Information Processing

A number of non-neural cells have been shown to exhibit memory, with respect to somatic position (Carlson, 1983; Chang et al., 2002; McCusker and Gardiner, 2014) or differentiation (Xiong and Ferrell, 2003), implemented via long-term stable changes in bioelectric state (Marder et al., 1996; Turrigiano et al., 1996; Rosen and Cohen, 2006) and transcriptional profile (Kragl et al., 2009; Wang et al., 2009). These are now beginning to be understood via physiological modeling and dynamical systems theory that views memories as attractors in transcriptional, bioelectric, or epigenetic state space (Huang et al., 2005; Cervera et al., 2015; Law and Levin, 2015).

Moving up in organization, several tissues have been suggested to exhibit memory. One is bone, which has many similarities to a neural network, both molecularly and functionally (Turner et al., 2002). For example, the neurotransmitter glutamate plays a role in cell-to-cell communication among bone cells. Glutamate of course is a key neurotransmitter for learning and memory in the hippocampus. Bone cells exhibit habituation (to repeated mechanical stimuli) and sensitization (to mechanical loading) – two of the most basic components of memory. Skull bones react quite differently to mechanical loading and hormones than do long bones, and it has been speculated that the past history of weight bearing imparts long-term cellular memory to the bone cell network, manifesting as differential responses to a variety of stimuli. A model involving long-term potentiation via the NMDA receptor has been proposed to explain memory of past stresses, and its subsequent influence over growth control, has been proposed (Spencer and Genever, 2003; Ho et al., 2005). Muscle comprises of some of the largest cells of animals, and also process, store and retrieve information via muscle-specific memory which can last from 15 years up to the entire lifetime in humans (Bruusgaard et al., 2010; Gundersen, 2016).

A most interesting set of studies have examined the phenomenon of cardiac memory. This is a clinically important pathway, in which specific changes of heartbeat pattern can persist stably (Otani and Gilmour, 1997; Goldberger and Kadish, 1999; Rosen and Plotnikov, 2002). This phenomenon has been modeled as a simple memory-like quantity that determines the relationship among the durations and amplitudes of action potentials (Otani and Gilmour, 1997). Most importantly, a specific mathematical model has been proposed for cardiac memory, taken after Hebbian plasticity in the brain (Chakravarthy and Ghosh, 1997; Zoghi, 2004).

The most recent addition to this body of work is the study of pancreas physiology (Goel and Mehta, 2013), which studied gap junctions (electrical synapses used for ionic communication in the brain, heart, and other organs), and their role in secretion of insulin from the pancreatic islets of Langerhans in response to glucose stimulation. Gap junctions synchronize oscillations of resting potentials among beta cells, and thus control insulin secretion. Past measurements of gap junctional conductance was unable to explain systemic properties, such as diminished junctional coupling in type-2 diabetes. In contrast to the prevailing tendency to focus on bottom-up views of the molecules involved and their interactions, Goel and Mehta viewed the process top–down, as a learning-like adaptation. Modeling gap junctions as links in a network of beta cells, subjected to homeostatic plasticity, they elucidated the system-level properties of this tissue, explaining why reductions in gap junction-mediated coupling in diabetes is necessary for an increase in blood insulin levels following hyperglycemia. It is not yet known if these mechanisms also underlie classical studies by Pavlov and others (Gantt, 1974, 1981; Gantt et al., 1987) on the classical conditioning of body organs to sugar, adrenaline, histamine, and other physiological stimuli.

We next consider larger-scale multicellular systems, at the level of organs or whole bodyplans (Levin, 2012b). Many species’ bodies exhibit pattern memory during regeneration (Baddour et al., 2012; Lobo et al., 2014), and even transplanted organs maintain spatial information, such as transplanted eyes which send out optical axons to penetrate the brain on the side corresponding to its former location in a donor animal (Koo and Graziadei, 1995). It should be noted that one challenge to multicellularity is the ever-present danger of cancer – defection of somatic cells from the anatomical goals of the organism toward more primitive “every man for himself” behavior of individual cells and tumors (Johnston et al., 1992; Vincent, 2012; Chen and He, 2016). The interplay between the tumor and host has been analyzed using game theory (Dingli et al., 2009; McEvoy, 2009), consistent with each being an autonomous system with internal and external information channels, goals, and functional capabilities. Control networks regulating cancer have been analyzed from the perspective of learning (Gyurko et al., 2013), which represents an interesting new area for further research. Interestingly, recent data implicate in carcinogenesis the same bioelectric mechanisms that orchestrate pattern regulation and keep cells away from tumorigenesis (Chernet B. and Levin, 2013; Chernet B.T. and Levin, 2013; Yang and Brackenbury, 2013; Bates, 2015).

## Somatic Pattern Memories: Non-Neural Bioelectricity

The first task of any animal body is to assemble the progeny of a fertilized egg cell into a specific 3-dimensional pattern during embryogenesis. Then comes the need to maintain anatomical integrity over the lifespan, despite individual cell senescence, injury, and neoplastic conversion. Thus, long before animals developed brains to execute adaptive behaviors, cells had to have ways to coordinate their activity in an exquisite ballet that self-assembles, and then continuously remodels and repairs, a complex anatomical form. Some animals (e.g., salamanders) can regenerate their limbs, eyes, jaws, hearts, and portions of the brain (Sanchez Alvarado and Tsonis, 2006). Mammals have reduced powers of regeneration, but deer regenerate antler bone (adding up to 1 cm per day) every year, while humans regenerate their livers, and children regenerate their fingertips. Tails grafted onto the sides of salamanders slowly remodel into limbs (a structure more appropriate to their new location), and mammalian embryos can be split in half or combined together, resulting in normal embryos (reviewed in (Mustard and Levin, 2014)). All of these capabilities require significant information storage and processing, and many take place prior to (or without) the presence of the CNS.

Embryogenesis, regeneration, and metamorphosis stop precisely when the correct anatomical shape has been produced; this is a process akin to goal-directed behaviors, in the sense that the system can pursue multiple paths toward the same (anatomical) goal state, can accommodate unpredictable external perturbations (is not hardwired but flexible), and rests when it is satisfied (can recognize when its goal is achieved). All of these examples show the remarkable information processing that cells carry out, in order to create and maintain specific shapes (Levin, 2012b). Analogously to how brains implement goal-seeking behavior via information processing, non-neural cell networks process information about current and future anatomical shape. While the brain operates muscles and glands in service of activity in ecological space, the computational processes of non-neural somatic networks control cell behaviors (differentiation, migration, proliferation) to optimize the body’s movement through morphospace (Stone, 1997; Rasskin-Gutman and Izpisua-Belmonte, 2004; Newman and Bhat, 2009).

A primary goal of developmental biology, synthetic bioengineering, and regenerative medicine is to learn to understand and control patterning networks, for applications in birth defects, organ regeneration, and cancer reprogramming (Ingber and Levin, 2007; Doursat et al., 2013). In particular it is crucial to tame the endogenous closed-loop pattern regulatory systems (flexible remodeling and regeneration pathways), as these offer the opportunity to exploit modularity to achieve needed changes in growth and form without micromanaging the details. What mechanisms underlie the ability of tissues to measure large-scale shape, detect deviations from a “remembered” correct target morphology, implement remodeling toward repairing that shape, and know when to stop (Levin, 2011)? Recent work has shown that as in the brain, these control networks make use of ion channels, gap junctions (electrical synapses), and neurotransmitters (Levin, 2012a; Tseng and Levin, 2013). A parsimonious hypothesis is that this is no coincidence, and that the brain learned its prodigious computational tricks from far more ancient pathways, co-opting developmental bioelectricity and optimizing it for the speed needed for behavior. While the brain operates on millisecond-scale bioelectric spiking, developmental bioelectricity involves steady, slow changes in ion fluxes, resting potentials, and electric fields.

A long history of work implicated bioelectric events in patterning (Jaffe, 1981; Nuccitelli, 2003; McCaig et al., 2005). Recent advances in molecular physiology have revealed that gap junctions, ion channels, and neurotransmitter pathway molecules – workhorses of cognitive processes in the CNS – are broadly expressed throughout the body, beginning prior to fertilization. Analogously to the brain, non-neural tissues continuously regulate resting potential (V_mem_) and local field potentials (extracellular electric fields), as well as regulate the movement of neurotransmitters among cells (Pullar, 2011; Bates, 2015).

Signaling mediated by bioelectric events plays a crucial, instructive role in pattern formation (Funk, 2013; Levin, 2014b). Ion channel-mediated changes in V_mem_ not only affects individual cell behaviors such as proliferation, differentiation, apoptosis, and migration (Sundelacruz et al., 2009), but also determines large-scale parameters such as organ size, shape, and axial patterning of the entire body (Beane et al., 2011; Perathoner et al., 2014). In a range of model systems, V_mem_ regulates the formation of the brain, eye, wing, and face, and controls patterning along the anterior-posterior and left-right axes during embryonic development (Levin et al., 2002; Dahal et al., 2012; Pai et al., 2015a). Moreover, experimental control of bioelectric gradients has enabled induction of regenerative ability in non-regenerative contexts (Tseng et al., 2010; Leppik et al., 2015), induced reprogramming of gut tissue into complete eyes (Pai et al., 2012), and normalized tumors (Chernet B. and Levin, 2013; Chernet B.T. and Levin, 2013). Electrical synapses (gap junctions, GJs) and neurotransmitters like serotonin are a key component of several patterning systems, having been implicated in embryonic left–right asymmetry, bone patterning, tumor suppression, and brain size control (Levin and Mercola, 1998; Iovine et al., 2005; Chernet et al., 2015; Pai et al., 2015a). As in the brain, these elements often work together, such as the bioelectrically controlled movement of serotonin through GJs during left-right patterning and control of nerve growth (Levin et al., 2006; Blackiston et al., 2015). The molecular pieces are now being identified, but the idea of neurotransmitters being ancient “pre-nervous” developmental signaling molecules is an old one (Buznikov and Shmukler, 1981).

**FIGURE 2:**
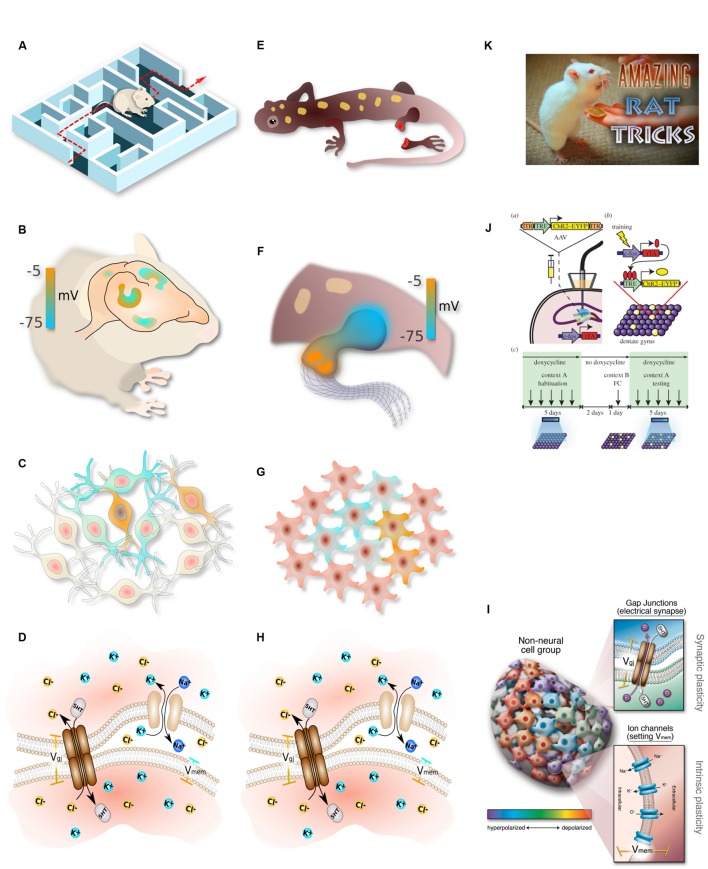
**Parallelism between neural and somatic computational systems**. Complex, flexible, goal-seeking behavior **(A)** is implemented by information processing in the brain **(B)**, which consists of networks of electrically communicating neural cells networks executing physiological circuits **(C)**, which operate because of electrically gated ion channel and electrical synapse proteins **(D)**. Similarly, large-scale goal-directed pattern remodeling and regeneration **(E)** occurs via bioelectric gradients that coordinate cell activity **(F)**, implemented by physiological circuits in non-neural cells **(G)** which operate because of the same set of ion channels and electrical synapses **(H)**. The behavior of these systems at the lowest level is achieved by regulating gap junction state and ion channel activity in specific cells **(I)**. Circuit activity is beginning to be tractable in both contexts using optogenetics **(J)**. In behavioral settings, the most effective path toward desired outcomes is to interact with the system at the highest level, rewarding for desired behavior **(K)**. This strategy remains to be tried in patterning contexts, where the current paradigm has been focused on bottom–up approaches and has not yet investigated the top–down strategies that have paid off so well for cognitive science. **(A–H)** drawn by Alexis Pietak. **(I,K)** drawn by Jeremy Guay of Peregrine Creative. **(J)** used with permission from Liu et al. (2014).

The analogy between the brain and somatic pattern control (**Figure 2**) makes several specific predictions. One is that ion channels, GJs, and neurotransmitters should play a role in development; this has been amply demonstrated by the identification of patterning channelopathies (Levin, 2013), functional experiments in regenerative and developmental biology (Stewart et al., 2007), and the teratogenic effects of numerous psychoactive drugs (Hernandez-Diaz and Levin, 2014). Another key prediction concerns the encoding of instructive information. In the brain, genetics establish the hardware – genes encode the available components and thus define the limits of cellular activity. However, the information content of the brain is not directly encoded by the genome, but rather arises dynamically through environmental stimuli (learning) and self-organizing dynamics of the electrochemical circuitry (plasticity). Is this the case in pattern formation as well?

Can “long term somatic memory” be edited, in the context of a wild-type genome, leading to a permanent change? A first example of this was shown in a different species of planaria (Nogi and Levin, 2005), where targeting GJs for just 48 hours in a chunk of tissue caused it to regenerate 2 heads – one at the former anterior end (normal), and one at the posterior-facing end (which would normally grow a tail). Strikingly, these 2-headed worms continue to regenerate as 2-headed when cut in subsequent rounds of regeneration, in plain water, months after the GJ blocking reagent is long gone from the tissue (Oviedo et al., 2010). The target morphology – the shape to which this animal regenerates upon damage – has been permanently re-written by temporarily editing the physiological network. This finding has clear similarity to plasticity [well-known to be exhibited by electrical synapses (Pereda et al., 2013)]: a brief induced change of GJ connectivity becomes stabilized to a long-term change (Levin, 2014a). This interaction between bioelectric activity and voltage-gated GJs makes developmental bioelectrical networks especially suitable as a labile yet stable memory medium (Palacios-Prado and Bukauskas, 2009). Another brain-like property exhibited in this effect is its holographic nature: in each round of cutting, the ectopic head (perhaps “epigenetically reprogrammed”) is removed, and a middle fragment of the gut still knows it must make 2 heads if cut. The patterning information is distributed non-locally throughout the network.

This field is advancing rapidly in its mechanistic details at the cellular level: the genetics of endogenous ion channels causing the gradients, the transduction mechanisms that control transcription after V_mem_ change, and the gene expression changes downstream of bioelectrical signaling are all becoming clear (Yang and Brackenbury, 2013; Pai et al., 2015b). Techniques, such as optogenetics (Adams et al., 2013, 2014), are allowing imposition of specific voltage patterns onto tissue *in vivo*. As in the brain, where optogenetics is used to insert memories directly into brains (Ramirez et al., 2013; Liu et al., 2014), these techniques will be crucial to learn to rewrite pattern memories during regeneration or embryogenesis. However, as in neuroscience, there is more than one level at which progress needs to be made. A mature understanding of the brain requires synthesis of data from people working on the genetics and biochemistry of specific neurotransmitter receptors and their downstream molecular signaling, with the insights of workers at the level of circuits, behavior, cognitive science, and psychology.

Classic work explored the extensive parallels between chemical gradients during development and signal processing in the visual system (Grossberg, 1978), and indeed early quantitative models of patterning (explaining self-regulatory features like proportion regulation) were based on visual system function (Hartline et al., 1956; Gierer and Meinhardt, 1972). More recent efforts include the notion of memory for position during regeneration (Chang et al., 2002; Kragl et al., 2009; Wang et al., 2009) and development (Beloussov, 1997) and for signaling hysteresis during development (Balaskas et al., 2012), excitable cortex memory models of pseudopod dynamics (Cooper et al., 2012), and neural network models of chemical signaling (Ling et al., 2013) (which showed formal isomorphisms between gene regulation networks and Hebbian learning in neural nets) (Watson et al., 2010; Ling et al., 2013). In addition to classical neuroscience concepts, more exotic group cognition models have been applied to patterning (Gunji and Ono, 2012), while a few recent studies investigated the decision-making and formal computational capabilities of RD systems – a chemical signaling modality often used to model morphogenesis (Adamatzky et al., 2003, 2008; Costello et al., 2009; Dale and Husbands, 2010, which is now known to be Turing-complete (Scarle, 2009) and support semantic interpretations (Schumann and Adamatzky, 2009). Despite these fascinating efforts to identify elements of cognitive-like processing in well-known elements of pattern formation, developmental biology is still firmly centered in a mechanistic perspective, seeking explanations in terms of pathways and not information (systems that know things and make decisions based on that understanding). However, it is crucial to note that attributing true knowledge and memory to biological systems is not mystical thinking – computational neuroscience shows us a clear proof of concept that information-level, cognitive approaches to cellular networks are viable, and in fact necessary, strategy for understanding a system at all of its salient levels.

Thus, neuroscience offers developmental biology more than just tools and molecular mechanisms: it offers a unique paradigm, otherwise unavailable to molecular and cell biologists, of the emergence of higher levels of organization that have both causal potency and experimental tractability. The field is in need of new formalisms and conceptual tools for linking the dynamics of physiological circuits with downstream patterning outcomes. Developmental biology is currently focused entirely in a bottom-up mode, with molecules being the preferred level of explanation. Neuroscience teaches us that we must look upward as well as downward, for emergent levels with their own rules and advantages (Friston et al., 2015). For example, training an animal to a particular complex behavior is far more efficient than attempting to elicit the same behavior by manipulating individual neurons in their brains. We now know that beneficial changes at the genetic and chemical levels can be induced by *cognitive* therapies – top–down control of tissue structure and function induced by specific thoughts and experiences. If patterning tissues are “primitive cognitive agents”, in the sense that they can be profitably understood as memory-bearing, information processing, goal-directed cybernetic systems (Pezzulo and Levin, 2015), then a whole new set of approaches becomes available for regenerative medicine. If we understood the bioelectric code, we could interact with it at these higher levels of organization, taking advantage of endogenous modularity and perhaps rationally controlling anatomical outcomes without having to micromanage molecular networks. In this field, cognitive science, unconventional computation, and developmental biology intersect. A fundamental open direction is the search for a computational pipeline to extract goal patterns from bioelectric state data, parallel to efforts to extract image data from brain measurements (Nishimoto et al., 2011). The flow of knowledge will likely not all be unidirectional: cracking the bioelectric code in patterning tissues is likely to in turn benefit fundamental neuroscience by showing, in perhaps a simpler context how to extract semantic content from bioelectrical cell states in the brain.

## Conclusion

**FIGURE 3:**
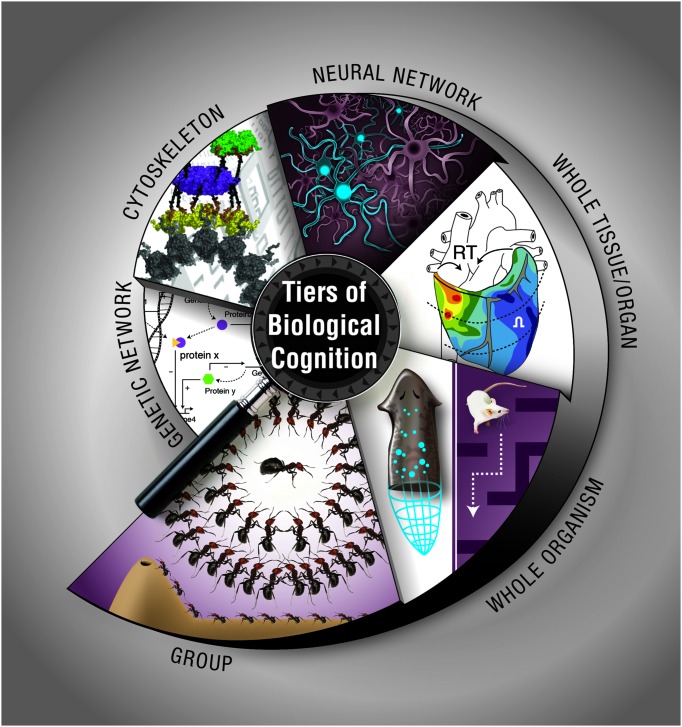
**Cognition at multiple, nested levels of biological organization**. Information processing, memory, and flexible decision-making is exhibited by biological systems such as chemical networks, cytoskeletal dynamics, neural networks, tissue, and organ physiological circuits, entire organisms during behavior or pattern formation, and groups of organisms in colonies. The cytoskeleton panel is used with permission from Craddock et al. (2012). Graphic created by Jeremy Guay of Peregrine Creative.

How does biological matter give rise to decision-making, memory, representation, and goal-directed activity? Implementation-independence is a core principle of computer science: an algorithm does what it does regardless of what kind of medium is implementing the steps. However, in the biological sciences, the study of memory and other cognitive functions has largely been the province of neurobiology, which studies the information processing and computational functions of one type of system: collections of neurons. Instead, we have surveyed a broad range of systems at various scales, from molecular to organismal, which have their own distinct ability to process information, make decisions, and achieve specific goal states (**Figure 3**). Neural-like computation, decision-making, and memory have been reported in sperm (Alvarez et al., 2013), amoebae (Zhu et al., 2013), yeast (Caudron and Barral, 2013), and plants (Gagliano et al., 2014), using ubiquitous mechanisms like cytoskeletal elements which appear to be also involved in neural information processing (Sahu et al., 2013). It is clear that neural networks have no monopoly on such functions. Remarkably, it is not only the positive (adaptive) cognitive functions that are widely conserved: some of the same illusions to which advanced brains’ perception and rational reasoning fall prey are being found in systems from slime molds to multi-animal colonies (Beekman and Latty, 2015; Sakiyama and Gunji, 2016).

McCulloch said “Why the mind is in the head? Because there, and only there, are hosts of possible connections to be performed as time and circumstance demand it” (McCulloch, 1951). Given the facts of protein, cytoskeletal, transcriptional, and bioelectric networks, it appears that many different media at various scales have the ability to form and rewire experience-dependent connections. The “dynamical hypothesis” (van Gelder, 1998) asks, what if the brain is better understood as a dynamical system, than a computational one? We invert this hypothesis, and ask what if some dynamical systems are better understood as cognitive agents? The appearance of memory and computation at many levels of biological organization suggests a fractal organization of cognitive subsystems within systems – molecular, cellular, tissue, and body-wide (**Figure 2**). This has been suggested in the brain [Smythies’ nested doll hypothesis, (Smythies, 2015)] but may indeed exist throughout the biological world. Whether each successive level of organization is in some sense smarter than the ones below it, or whether structures derive their cognitive powers from those of lower levels, remains to be discovered. It should be noted, however, that even in advanced brains, the relationship between cognitive capacity and biological structure is not trivial to pin down, as shown by the occasional example of potent function in the presence of severe structural deficits (Lorber, 1978, 1981; Nahm et al., 2012).

The hypothesis of nested, widely prevalent cognitive layers suggests a rich research program, including: (1) the development of improved methods for reading/writing bioelectrical state information into somatic tissues and sculpting non-neural bioelectric circuits (optogenetics beyond excitable cells and in the synthetic biology of gap junction and neurotransmitter signaling; Adams et al., 2013), (2) continued work on cracking the bioelectric code (bioelectric state information maps onto the topology of various patterning outcomes in tractable model systems such as planaria; Tseng and Levin, 2013), (3) formulation and testing of quantitative, molecular models of LTP, habituation, sensitization, plasticity, and higher-order learning applied to protein interaction networks, gene regulatory circuits, cytoskeletal dynamics, and cell behavior during morphogenesis, (4) use of reagents that impact cognition (hallucinogens, anesthetics; Kawamoto et al., 2005), stimulants, nootropics/cognitive enhancers, etc.) in cellular, developmental, and regenerative patterning assays to probe conservation of pathways between neuroscience and morphogenesis, (5) creation of larger-scale computational models of regeneration and functional experiments in morphogenesis based on goal-seeking and error minimization algorithms with molecularly specified metrics (Slack, 1980; Chao et al., 2008), (6) exploration of molecular models of cognitive concepts (attention, autism spectrum, sleep, visual illusions/hallucinations, addiction) in specific patterning and mispatterning contexts, (7) bioengineering platforms that reward and punish *in vitro* patterning systems for specific changes in growth and morphogenesis (instrumental learning and top–down control of shape in developmental or regenerative contexts), and (8) a mechanistic investigation of the mechanism of persistence of memories through massive brain regeneration, which is likely to reveal the interface between somatic and neural memories (Blackiston et al., 2008; Shomrat and Levin, 2013).

We have avoided here the thorny issues of philosophy of mind that arise from trying to define exactly under what conditions words like “knowledge” are appropriate, in favor of an intentional stance-like pragmatic, engineering approach grounded in cybernetics. The coverage of cognitive terms across biology can expand to the extent that information-centered approaches are shown to be effective in predicting and controlling the behavior of biological systems. The practical implications for biotechnology, unconventional computation, and regenerative medicine are enormous. Equally likely, the lessons we learn from unconventional cognitive systems will deeply impact our most basic understanding of how mind emerges from the brain.

## Author Contributions

ML and FB both contributed sections to the review according to their specialties. Both provided novel ideas, edited each other’s text, and prepared the finished product.

## Conflict of Interest Statement

The authors declare that the research was conducted in the absence of any commercial or financial relationships that could be construed as a potential conflict of interest.
